# Brain radiotherapy and anlotinib control primary cardiac angiosarcoma with metastases: A case report

**DOI:** 10.1097/MD.0000000000037914

**Published:** 2024-04-26

**Authors:** Ying-Ying Ma, Zhi-Ke Li, Zi-Yi Liao, Yang Peng, Li Zeng, Dai-Yuan Ma

**Affiliations:** a Department of Oncology, Affiliated Hospital of North Sichuan Medical College, Nanchong, China.

**Keywords:** anlotinib, brain metastases, immunotherapy, primary cardiac angiosarcoma, radiotherapy

## Abstract

**Rationale:**

Primary cardiac angiosarcoma (PCA) is a rare and fatal disease with a poor prognosis. Whether the survival of PCA patients can be prolonged with additional treatment following complete surgical excision is controversial.

**Patient Concerns:**

In this case study, a 52-year-old male complained of chest tightness and pain for 7 days before admission into the hospital. Subsequently, he revisited the hospital because of dizziness and headache.

**Diagnoses:**

Initially, the patient was diagnosed with PCA in the right atrium by thoracic computed tomography (CT). Palliative resection identified brain, lung, and liver metastases.

**Intervention:**

The patient accepted multimodal combination therapy, including first-line chemotherapy and then second-line anlotinib concurrent with brain radiotherapy and immunotherapy.

**Outcome:**

Although anlotinib combined with brain radiotherapy controlled the growth of intracranial lesions, progression-free survival (PFS) was only 5 months, and the overall survival (OS) was only 12 months.

**Lesson:**

The treatment for metastatic PCA needs an in-depth exploration.

## 1. Introduction

Primary cardiac angiosarcoma (PCA) is a rare cardiac tumor comprising approximately 33% of all primary malignant cardiac neoplasms.^[1]^ PCA exhibits poor prognosis in patients and is characterized by high invasiveness and rapid progression of the tumor. Approximately 66% to 89% of patients exhibit PCA metastasis, commonly in the lung and bone and occasionally in the liver and brain, at the time of diagnosis.^[2–4]^ Surgical resection is the preferred method for tumor management. The median survival time is 6 months for patients with primary cardiac malignant tumors^[5]^ and 14 and 6 months for those with operable and inoperable tumors, respectively.^[6]^ Other management strategies include chemotherapy, radiotherapy, immunotherapy, targeted therapy, or a combination of these approaches. Currently, a consensus for the treatment of PCA is lacking.

Here, we report a case of right atrial angiosarcoma with metastasis. The patient was treated with multimodal combination therapy. Anlotinib combined with brain radiotherapy effectively controlled intracranial lesions. Consequently, the progression-free survival (PFS) was 5 months, and the overall survival (OS) was 12 months, indicating that combination therapy could be effective for PCA, although an in-depth exploration of the approach is imperative.

## 2. Case presentation

**Figure 1.**
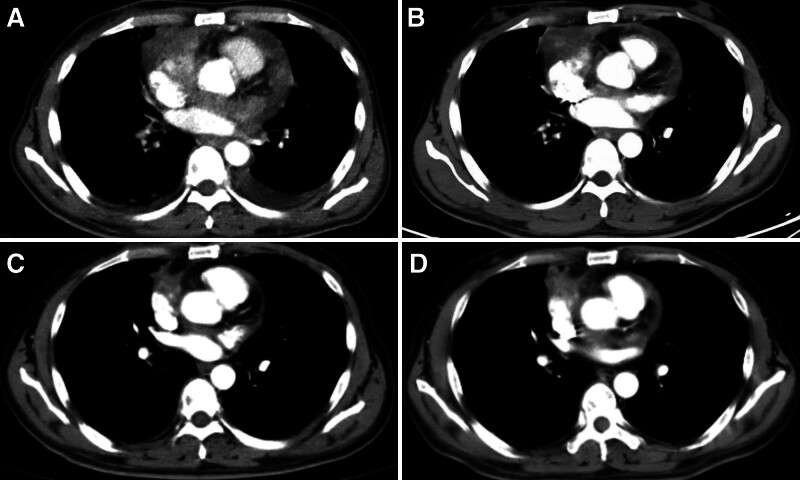
CT of PCA. (A) Before resection of the heart tumor (about 3.5 cm × 3.2 cm) on May 18, 2021. (B) Recurrent lesions after operation on March 31, 2021. (C) After 2 cycles of anlotinib on August 27, 2021, PR was evaluated. (D) After 4 cycles of anlotinib on October 24, 2021, PD was evaluated. CT = computed tomography, PCA = primary cardiac angiosarcoma, PD = progressive disease, PR = partial response.

**Figure 2.**
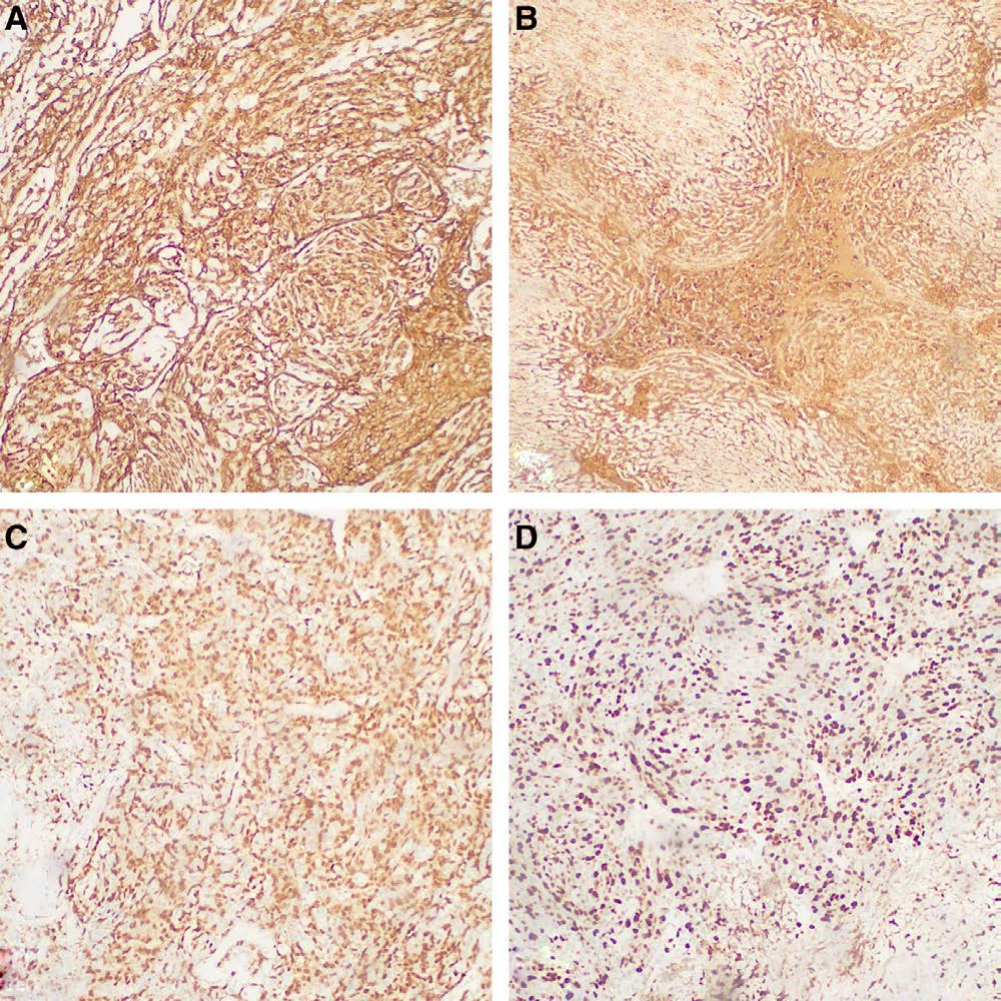
Immunohistochemistry of the tumors. (A) Positive for CD31, ×100. (B) Positive for CD34, ×100. (C) Positive for ERG, ×100. (D) Positive for Ki-67 (about 40%), ×100.

**Figure 3.**
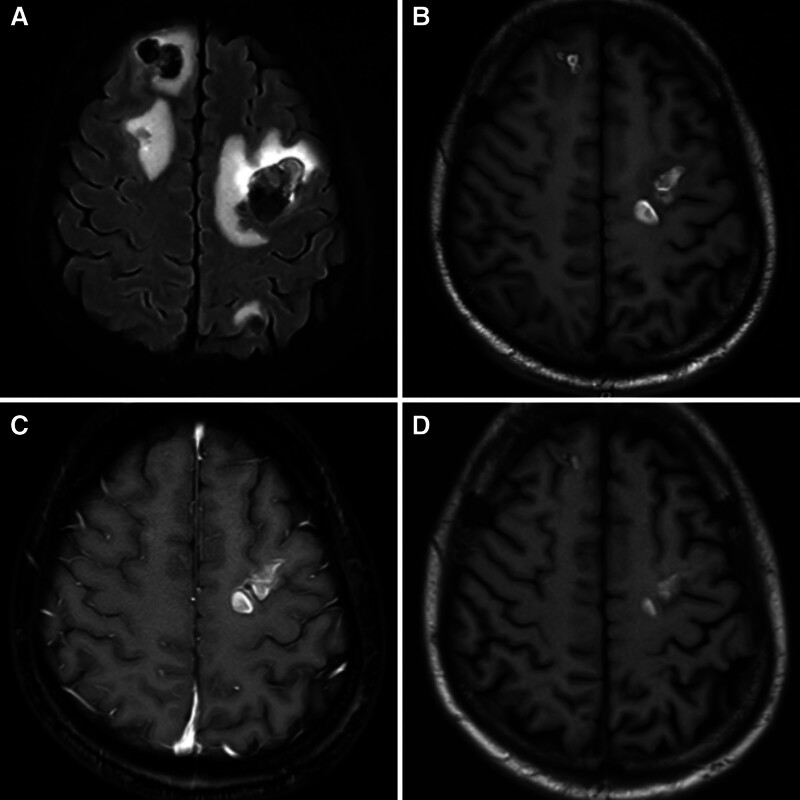
MRI of the brain lesions. (A) Before brain radiotherapy on May 19, 2021. (B) After anlotinib synchronized with brain radiotherapy on July 5, 2021. (C) After 2 cycles of anlotinib synchronized with brain radiotherapy on August 30, 2021. (D) After 4 cycles of anlotinib synchronized with brain radiotherapy on October 24, 2021. MRI = magnetic resonance imaging.

A 52-year-old male was admitted to the Department of Oncology in our Affiliated Hospital of North Sichuan Medical College (Nanchong, China) in January 2021 because of chest tightness and pain for 7 days. Heart ultrasound showed fluid collection in the pericardium. Thoracic computed tomography (CT) also revealed a malignant tumor in the right atrium (about 3.5 cm × 3.2 cm) with a small amount of pleural effusion (Fig. 1A). The patient underwent palliative resection on February 2, 2021. The tumor was pathologically identified as angiosarcoma due to the expression of tumor-positive markers: CD31, CD34, ERG, and Ki-67 (Fig. 2). One month later, the patient developed a cough and hemoptysis. Chest CT showed metastases in the pericardium, right upper lobe of the lung, thoracic vertebra, and right pleura (Fig. 1B). The chemotherapy cycle consisted of an ifosfamide and epirubicin regimen (epirubicin 100 mg d1-2 + ifosfamide 3.0 g d1-5) beginning on April 16, 2021. The single cycle resulted in limb edema and myelosuppression (degree IV). Recombinant human granulocyte colony-stimulating factor (rhG-CSF, 150.0 mg d1-6) was administered on April 24, 2012, following which the patient was discharged. On May 19, 2021, the patient revisited our hospital because of dizziness and headache. Abdominal CT revealed multiple lesions on the liver, while magnetic resonance imaging (MRI) of the head detected metastases in the brain, mainly in the left parietal lobe (Fig. 3A). Brain radiotherapy [planning target volume: 30 Gy/10 Fx, planning gross tumor volume: 52 Gy/12 Fx] was synchronized with anlotinib (10 mg, d1 to 14, q3w) on June 2, 2021, following which the symptoms were significantly relieved; the evaluation endpoint was partial response (PR) assessed according to the Response Evaluation Criteria in Solid Tumors, version 1.1 (Fig. 3B). The patient accepted 4 cycles of chemotherapy plus targeted therapy (paclitaxel 210 mg d1, anlotinib 10 mg d1-d14, q3w) starting on July 4, 2021. After 2 cycles, PR was estimated on August 27, 2021 (Fig. 3C), and after completion of the 4 cycles, metastatic lesions in the pericardium, lung, thoracic vertebra, and liver had progressed, while the brain lesions had decreased. Comprehensive evaluation showed progressive disease (Fig. 3D). During this period, the patient developed hemoptysis, due to which the treatment of anlotinib was temporarily discontinued on October 20, 2021. The patient then underwent 2 cycles of immunotherapy with tirelizumab (an antibody against programmed cell death protein 1) starting on October 27, 2021. The patient self-administered anlotinib but died on December 28, 2021, with an OS of 12 months.

## 3. Discussion

Cardiac neoplasm was first reported in 1559 by Dr Realdo Colombus, and the clinical diagnosis of primary cardiac sarcoma was first reported in 1934.^[7]^ Approximately 90% of PCAs occur in the right atrium, growing rapidly, infiltrating the myocardium, and invading the vena cava, tricuspid valve, atrium, and other adjacent structures.^[8]^ Pericardial effusion can also be detected in patients with PCAs, and about 50% of patients present complications of pericardial tamponade. These pathological changes lead to chest pain, edema, pulmonary arterial hypertension, congestive heart-failure, and other related symptoms.^[9,10]^ The patient in this case study repeatedly presented with chest tightness, pain, and pericardial effusion, followed by multiple systemic metastatic lesions in the lung, liver, brain, and bone. This phenomenon indicated poor survival, which was consistent with the previous reports.

The main diagnostic methods for PCA include echocardiography, CT, and MRI. Primary and secondary cerebral angiosarcomas are observed as well-circumscribed tumors with hemorrhage and surrounding edema on CT or MRI.

After resection of the heart tumor, radiotherapy or the previously administered drugs active in the central nervous system could prevent brain metastasis. However, after the tumor has progressed to the brain, drugs cannot penetrate the central nervous system effectively,^[11]^ thereby rendering poor patient survival. In the review by Drosos et al,^[12]^ the median OS of brain metastasis from angiosarcoma was 7.2 months, while the PFS was 1.5 months; the lesions originating from the heart were worse than those from other organs. Although the disease had progressed rapidly, the PFS of the patient presented here was 5 months after anlotinib + brain therapy. The intracranial lesions were found to be controlled adequately until the patient death, as assessed by PR. This finding indicates that brain radiotherapy combined with anlotinib is an optimal method to control brain metastases from PCA.

Reportedly, combination therapy, including palliative surgery, radiotherapy, adjuvant chemotherapy, and immunotherapy, can improve survival for some PCA patients.^[13]^ Li et al^[14]^ reported a patient with metastatic cardiosarcoma who received chemoradiotherapy (epirubicin 10 mg, qd, ifosfamide 5 g, qd) and immunotherapy (pembrolizumab 100 mg, qd) for 4 cycles and radiotherapy for rib metastases (20 Gy/5 fx), which resulted in significant regression of the primary and metastatic lesions. Fang et al^[15]^ also reported a patient with metastatic cardiosarcoma (with no brain lesions) who was treated with chemotherapy (gemcitabine) combined with docetaxel for 8 cycles, followed by radiotherapy synchronous with 5 cycles of anlotinib; the OS was 23 months. These findings indicate that radiotherapy plus anlotinib is efficacious in PCA. In the case presented here, brain radiotherapy combined with anlotinib controlled the intracranial lesions but did not translate into OS benefits. Thus, PCA with brain metastasis may have low survival, and the clinical value of anlotinib plus radiotherapy in these patients requires further investigation.

## 4. Conclusion

PCA is a rare but fatal disease with poor prognosis, especially in the presence of brain metastasis. Anlotinib and anlotinib combination therapy are beneficial for brain metastasis and may have a wide application, but an in-depth exploration is essential.

## Acknowledgments

We thank Medjaden Inc. for its assistance in the preparation of this manuscript.

## Author contributions

**Conceptualization:** Dai-Yuan Ma.

**Data curation:** Ying-Ying Ma, Zhi-Ke Li.

**Formal analysis:** Li Zeng.

**Funding acquisition:** Dai-Yuan Ma.

**Investigation:** Li Zeng.

**Project administration:** Dai-Yuan Ma.

**Supervision:** Dai-Yuan Ma.

**Writing – original draft:** Ying-Ying Ma, Zhi-Ke Li.

**Writing – review & editing:** Zi-Yi Liao, Yang Peng, Dai-Yuan Ma.

## References

[1] Liu C Zhao Y Yin Z. Right atrial epithelioid angiosarcoma with multiple pulmonary metastasis confirmed by multimodality imaging-guided pulmonary biopsy: a case report and literature review. Medicine (Baltimore). 2018;97:e11588. 30045289 10.1097/MD.0000000000011588 PMC6078731

[2] Koo J Knight-Perry J Galambos C. Pediatric metastatic cardiac angiosarcoma successfully treated with multimodal therapy: case report and review of literature. J Pediatr Hematol Oncol. 2021;43:e203–6. 31725539 10.1097/MPH.0000000000001674

[3] Jain A Simon S Elangovan I. (18)F-fluoro-deoxyglucose positron emission tomography-computed tomography in initial assessment and diagnosis of right atrial angiosarcoma with widespread visceral metastases: a rare case report and review of the literature. Indian J Nucl Med. 2015;30:51–4. 25589807 10.4103/0972-3919.147541 PMC4290067

[4] Waness A Batoon AA Mirza I. Elusive cardiac angiosarcoma in a young pregnant female: rare presentation with fatal outcome. Cardiol Res. 2015;6:292–6. 28197244 10.14740/cr402w PMC5295525

[5] Antonuzzo L Rotella V Mazzoni F. Primary cardiac angiosarcoma: a fatal disease. Case Rep Med. 2009;2009:591512. 19724650 10.1155/2009/591512 PMC2731464

[6] Jang Y Kim J Shim JW. Primary cardiac angiosarcoma: a prolonged response to surgical resection followed by concurrent chemoradiotherapy with docetaxel. Springerplus. 2016;5:648. 27330914 10.1186/s40064-016-2248-8 PMC4870531

[7] Barnes AR Beaver DC Snell AM. Primary sarcoma of the heart: report of a case with electrocardiographic and pathological studies. Am Heart J. 1934;9:480–91.

[8] Silver M Gotlieb AI Schoen FR. Cardiovascular Pathology. 3rd Edition. Philadelphia: Churchill Livingstone. 2001.

[9] Leja MJ Shah DJ Reardon MJ. Primary cardiac tumors. Tex Heart Inst J. 2011;38:261–2. 21720466 PMC3113129

[10] Grebenc ML Rosado de Christenson ML Burke AP. Primary cardiac and pericardial neoplasms: radiologic-pathologic correlation. Radiographics. 2000;20:1073–103; quiz 1110. 10903697 10.1148/radiographics.20.4.g00jl081073

[11] España P Chang P Wiernik PH. Increased incidence of brain metastases in sarcoma patients. Cancer. 1980;45:377–80. 6243247 10.1002/1097-0142(19800115)45:2<377::aid-cncr2820450231>3.0.co;2-5

[12] Drosos E Kalyvas A Komaitis S. Angiosarcoma-related cerebral metastases: a systematic review of the literature. Neurosurg Rev. 2020;43:1019–38. 31165296 10.1007/s10143-019-01127-y

[13] Cao J Wang J He C. Angiosarcoma: a review of diagnosis and current treatment. Am J Cancer Res. 2019;9:2303–13. 31815036 PMC6895451

[14] Li X Lan L Hu H. Case report: Primary cardiac angiosarcoma with multiple metastases. Front Cardiovasc Med. 2022;9:941967. 35966523 10.3389/fcvm.2022.941967 PMC9366849

[15] Fang X Zheng S. Primary cardiac angiosarcoma: a case report. J Int Med Res. 2021;49:3000605211033261. 34433329 10.1177/03000605211033261 PMC8404650

